# MicroRNAs in innate immune responses and autoimmune diseases

**DOI:** 10.1186/ar3976

**Published:** 2012-09-27

**Authors:** EKL Chan

**Affiliations:** 1 University of Florida, Health Science Center, Gainesville, FL, USA

## 

MicroRNAs (miRNAs) are ~22 nucleotide single-stranded RNAs that regulate the stability of target messenger RNAs (mRNAs) via selective binding to specific sites at the 3'-UTR. This triggers repression in translation and mRNA degradation. It has been estimated that ~60% of all mRNAs are under the control of miRNAs. Among the known hundreds of miRNAs, some are considered master regulators controlling either a single or multiple cellular pathways. Some miRNAs are known to affect development and cell differentiation, while others are implicated in immunity and autoimmune diseases. A very interesting example is miR-146a, which has been reported to be downregulated in systemic lupus erythematosus and upregulated in rheumatoid arthritis. This miRNA plays a dominant role in the regulation of the innate immune responses. The overexpression or underexpression of miRNAs can influence specific targets and pathways, leading to autoimmune disease phenotypes, and this is also supported by some *in vivo *studies. Targeting miRNAs could represent a valid future therapeutic option for autoimmune diseases. This discussion will focus on the current understanding in the function of miR-146a in endotoxin tolerance and cross-tolerance, and how it may contribute to modulate the overproduction of known pathogenic cytokines.

